# The significance of cerebellar contributions in early-life through aging

**DOI:** 10.3389/fncom.2024.1449364

**Published:** 2024-08-27

**Authors:** Jessica L. Verpeut, Marlies Oostland

**Affiliations:** ^1^Department of Psychology, Arizona State University, Tempe, AZ, United States; ^2^Swammerdam Institute for Life Sciences, University of Amsterdam, Amsterdam, Netherlands

**Keywords:** cerebellum, Alzheimer's disease, neurodevelopment, aging, Purkinje cell, neural circuits, sex differences

## Introduction

**Figure 1:**
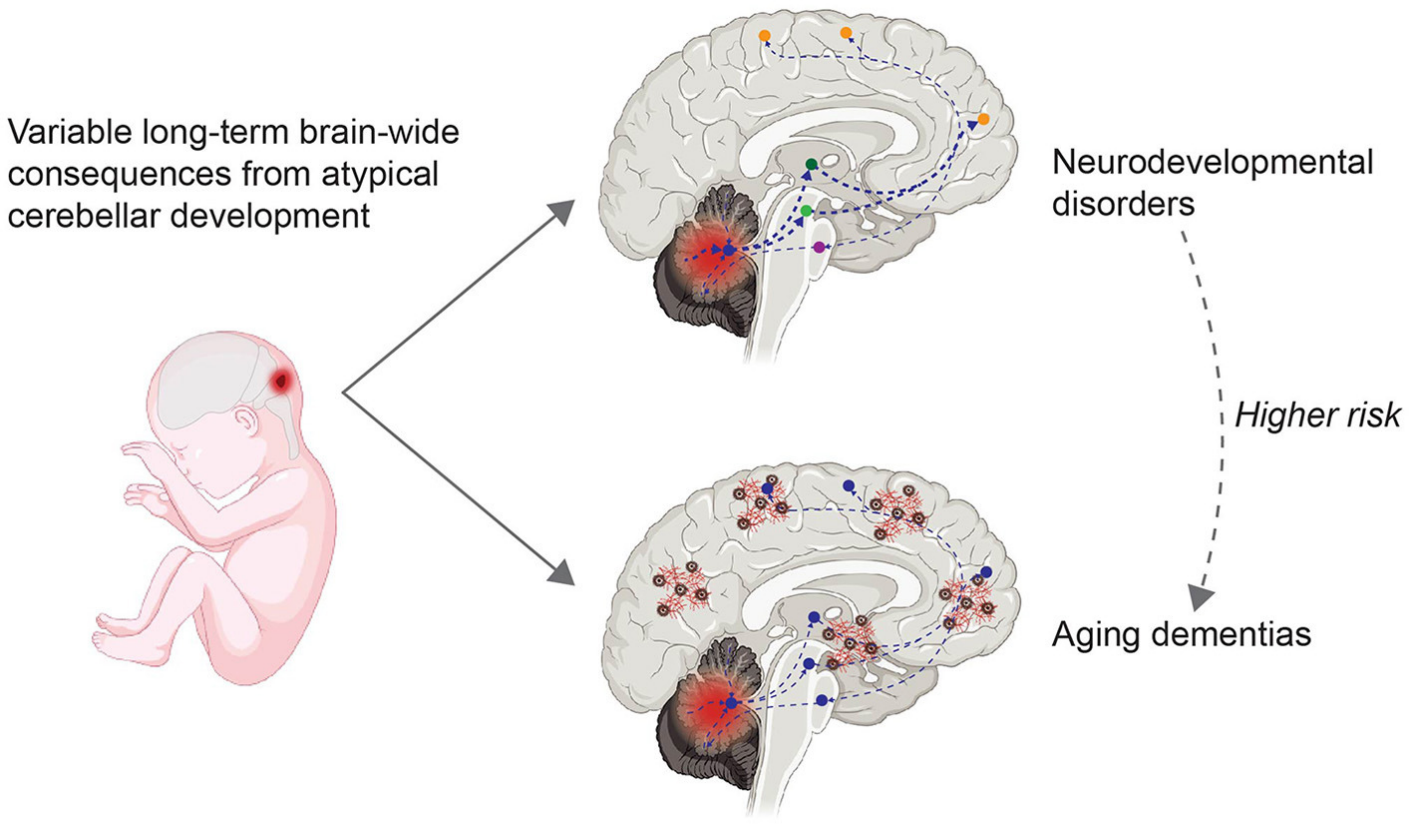
Variable long-term consequences of atypical cerebellar development. Atypical cerebellar development, i.e. due to perinatal injury to the cerebellum (**left**), can have long-term brain-wide consequences due to bidirectional connections between the cerebellum and neocortical areas (**right**). Information from the cerebellar cortex flows via the cerebellar nuclei (indicated by a blue circle) and subcortical regions such as the thalamus (dark green) or VTA (light green) to frontal regions (orange), and information from the frontal region flows back to the cerebellum via the pons (purple). Dashed lines indicate altered connections. People diagnosed with neurodevelopmental disorders (top right) have an increased risk of developing aging dementias (bottom right). Red and brown cortical elements in the bottom right indicate amyloid beta plaques and tau tangles, characteristics of aging dementias. Created with BioRender.com.

Cerebellar microanatomy has inspired many computational models that contribute to both motor and nonmotor function. Key contributors to understanding cerebellar function, including Marr, Albus, and Ito, described error signals within the cerebellum that drive synaptic plasticity and contribute to motor learning (Marr, 1969; Albus, 1971; Ito, 1972). These error signals within the cerebellum are the result of comparing motor output with sensory feedback from the environment. The resulting predictions and corrections for movement are transferred to output regions via bidirectional connections between the cerebellum and cortical regions. The establishment of these pathways in development and plasticity occurs early in life. Gross cerebellar development including the migration, growth, and maturation of neurons continues after birth, up to 3 weeks postnatally in rodents and one year postnatally in humans (Ten Donkelaar and Lammens, 2009). Total cerebellar volume continues to expand until adolescence, peaking earlier for girls (around 12 years of age) than for boys (around 16 years of age), similar to cerebral volume (Tiemeier et al., 2010). However, compared to other brain regions involved in aging such as the hippocampus, the aging cerebellum and its contribution to neurodegenerative diseases has received less attention. Recent evidence has shown that cerebellar connections become dysregulated in aging and may contribute to cognitive decline in Alzheimer's disease (AD), schizophrenia, and Parkinson's disease (Andersen et al., 2012; Douaud et al., 2014; Gellersen et al., 2021). Here, we argue that there are long-term consequences of atypical cerebellar development (Figure 1), and that patterns of cerebellar abnormality may be different between males and females. Furthermore, different patterns of cerebellar abnormality may explain both the diversity and variation of phenotypes in neurodevelopmental disorders and aging dementias. In this review we will largely focus on the prefrontal cortex, due to the significance of this region in cognitive and social behavior. Understanding how cerebellar-cerebrum networks break down in disease pathologies can lead to further understanding of neural networks and improve future therapeutics.

## Brain-wide cerebellar circuits can explain diversity in cerebellum-mediated phenotypes

The cerebellum projects disynaptically to all of neocortex as well as subcortical areas, including regions important for cognition and affective behavior, such as the medial prefrontal cortex (mPFC), anterior cingulate cortex (ACC), parietal cortex, and amygdala (Sveljo et al., 2014; Onuki et al., 2015; Pisano et al., 2021; Zhu et al., 2023). The largest cells of the cerebellar cortex, Purkinje cells, integrate information from ~70 billion neurons in the cerebellar cortex (Lange, 1975). GABAergic Purkinje cells are the only neuron type whose axon leaves the cerebellar cortex to synapse onto cells in the cerebellar nuclei (CN), therefore modulating excitatory CN output to the rest of the brain (Houck and Person, 2014; Judd et al., 2021). In addition, cerebellar output is transferred from the cerebellar cortex to the cerebellar nuclei in unique functional microzones which are organized in a largely lobule-dependent manner (Schweighofer, 1998; Kostadinov et al., 2019; De Zeeuw, 2021). The CN have direct connections with the thalamus, the ventral tegmental area (VTA) and dozens of different nuclei across the brain, and thereby indirect connections with the rest of the brain (Hoshi et al., 2005; Bostan and Strick, 2010; Watabe-Uchida et al., 2012; Hunnicutt et al., 2014; Houck and Person, 2015; Carta et al., 2019; Sathyamurthy et al., 2020; Kebschull et al., 2024; Washburn et al., 2024). In this way, cerebellar output can have widespread influence on the rest of the brain, including cognitive- and socially-associated brain regions.

In humans, Purkinje and granule cells are born and migrate starting at 7 weeks of age (van der Heijden and Sillitoe, 2021), then undergo multiple phases of plasticity and pruning prior to maturation in adulthood. During early development, neurons are sensitive to environmental stimuli as they undergo growth, differentiation, and pruning (Huttenlocher and Dabholkar, 1997). Cerebellar long-range synaptic connectivity, plasticity, and functional activity may be particularly vulnerable in early-life. Hence, there is a need to define causal links between cerebellar plasticity, circuit development, and behavior. Mouse models of social and cognitive deficits exhibit altered expression of signaling molecules and inhibitory circuits resulting in disruption of the excitatory/inhibitory (E/I) balance, reduced cortical connectivity, reduced gray matter, shorter axons, and reduced spine numbers (Schofield et al., 2011; Portera-Cailliau, 2012; Stoya et al., 2014; Shapiro et al., 2017). However, the role of cerebellar-thalamic-cortical projections in later stages of the lifespan has not been characterized and the distinct contributions of cerebellar signaling input on cerebral structure is unknown. Similarly to how the role of the cerebellum in shaping development is likely to be region-specific (Stoodley et al., 2017), different patterns of cerebellar abnormality leading to impaired anatomical and/or physiological connections with the neocortex could explain both the diversity and variation of phenotypes in neurodevelopmental disorders and aging dementias (Badura et al., 2018; Gellersen et al., 2021; Oostland et al., 2022; Bernard et al., 2023). Yet, specific details of the different patterns of cerebellar abnormality within cerebellar-cerebral pathways leading to neurodevelopmental and/or neurodegenerative disorders remain an open question.

## Unique cerebellar contributions to behavior between young and adult phenotypes

The cerebellum is among the most-often-reported sites of abnormality in a variety of developmental disorders, including autism spectrum disorder (ASD) and attention deficit hyperactivity disorder (ADHD) (Courchesne, 1997; Courchesne et al., 2001; Carper et al., 2002). Cerebellar injury in newborns, most commonly a stroke, leads to an overall 36-fold risk of an ASD diagnosis by age two (Limperopoulos et al., 2007; Wang et al., 2014). However, adult cerebellar injury does not cause ASD (Limperopoulos et al., 2007). Instead, it has been suggested that the cerebellum interprets and processes sensory stimuli (Baumann et al., 2015), and individuals without normal sensory stimuli in early life are at risk for developing ASD endophenotypes (Chugani et al., 2001; Le Mare and Audet, 2006). One likely target of cerebellar influence is the neocortex, which is highly plastic during development and whose circuitry is perturbed in ASD (Kalia, 2008; LeBlanc and Fagiolini, 2011). Neurons responsible for polysynaptic long-distance communication between the cerebellum and neocortex are highly plastic in early development, including Purkinje cells and cortical interneurons (Hoxha et al., 2016; Rupert and Shea, 2022), suggesting that starting in early life, the cerebellum may influence the development of cognitive and social capacities.

Disruptions in both long-distance and local brain signaling are thought to shape ASD, leading to gross anatomical deficits, including brain size and spine density. Deficits in cell migration, unbalanced excitatory-inhibitory networks, and improper synapse formation and pruning may contribute to these phenotypes (Johnston, 2004; Paus et al., 2008; Takeuchi, 2011; Thomas et al., 2011). During early postnatal development, an increase in synaptic growth, brain glucose metabolism, and gray matter density is followed by a significant pruning event (Andersen, 2003). This sensitive period of development allows for enhanced plasticity and shaping of neural circuits. Excitatory neural output from the cerebellar nuclei on nonmotor neocortical regions may shape dendritic arborization and spine formation to establish and maintain synaptic connections. We propose that the cerebellum shapes synapsing thalamic connections and disruptions during neural development alter growth and maturation of distal forebrain circuits.

The role of the cerebellum in aging is nearly completely unexplored and while the cerebellum does demonstrate aging symptoms, such as thinning of the cerebellar cortex and deposition of amyloid (Aranca et al., 2016; Jacobs et al., 2018; Miguel et al., 2021; Frangou et al., 2022; Toniolo et al., 2023; Arleo et al., 2024), the cerebellum demonstrates aging neuroprotective properties as well. Cerebellar injury to adults results in Cerebellar Cognitive Affective Syndrome and symptoms are distinct from ASD (Schmahmann, 2004, 2013). Indeed the cerebellum has been hypothesized to have neuroprotective properties (Gellersen et al., 2021) that may slow aging, including less age-related DNA methylation (Horvath et al., 2015), mitochondrial DNA deletions (Corral-Debrinski et al., 1992), oxidative damage (Mecocci et al., 1993), and resistance to soluble amyloid-beta (Kim et al., 2003; Yadollahikhales and Rojas, 2023). Unlike the cerebellar cortex, the CN displays more abundant neurodegenerative pathology including tau accumulation (Coakeley et al., 2017; Ikeda et al., 2017; Whitwell et al., 2017; Hammes et al., 2018), iron deposition in Friedrich's ataxia (Akhlaghi et al., 2012; Dogan et al., 2016; Vavla et al., 2018), cell atrophy in frontotemporal dementia (Braak and Braak, 1998; Braak et al., 1999; Rajan et al., 2015; Kneynsberg et al., 2017), Lewy body accumulation (Wu and Hallett, 2013; Bernard et al., 2023; Sivaranjini and Sujatha, 2024), and changes in cell cycle expression (Chen et al., 2010; Beeraka et al., 2022; Ali et al., 2024). It is thought that glia play an important role in aging dementias, but while the cerebellar cortex has more neurons than the rest of the brain, there are lower concentrations of glia. Bergmann glia or Golgi epithelial cells, astrocytes unique to the cerebellum, are situated predominantly in the Purkinje cell layer and are critical for neural migration in early development (Rakic and Sidman, 1973a,b; Xu et al., 2013). Evidence suggests that Bergmann glia support Purkinje cells and modulate structural plasticity, but how these may contribute or protect the cerebellum in aging is unknown (Matsui and Jahr, 2003; Coesmans et al., 2004; Matsui et al., 2005). There are multiple questions of the aging cerebellum to answer and understanding these biological mechanisms may explain contributions or resilience of the cerebellum to aging-related diseases.

## Sex differences in cerebellar anatomy and influence on aging

Is the cerebellum sexually differentiated? Along with the neocortex, the cerebellum is larger in males, with additional variations including differences in gray and white matter quantity (Ruigrok et al., 2014). In addition, males show stronger connections between the left cerebellar hemisphere and contralateral cortex (Tiemeier et al., 2010; Wheelock et al., 2019). In ASD, female brains have cortico-cerebellar hyperconnectivity, while males have a hypoconnective pattern (Smith et al., 2019). Neuroendocrine hormones, such as estrogen and progesterone, also regulate cerebellar cortical formation. Within the cerebellum, Purkinje cells express the estrogen receptors (ER) ERα and ERβ, the aromatase gene (Cyp19a), and intranuclear receptors for progesterone throughout life (Ukena et al., 1998; Sakamoto et al., 2001, 2002, 2003; Ikeda and Nagai, 2006; Hoffman et al., 2016; Hedges et al., 2018). Purkinje cell development may be modulated by estradiol through prostaglandin E2 (PGE2) and aromatase activity (Hoffman et al., 2016). During neonatal life, Purkinje cells synthesize progesterone from cholesterol *de novo* (Furukawa et al., 1998; Ukena et al., 1998, 1999). Progesterone increases Purkinje cell dendritic growth, spinogenesis, and synaptogenesis (Sakamoto et al., 2001, 2002). Some of the progesterone Purkinje cells metabolize may be converted to allopregnanolone, which has been shown to be neuroprotective and even delay neurodegeneration for Purkinje and granule cells (Griffin et al., 2004). These neuroendocrine factors may have unique functions across the lifespan. In development the cerebellum has been implicated in ASD, which is more commonly diagnosed in males than females (3:1) (Loomes et al., 2017). In aging, AD is twice as common in females than in males (Seshadri et al., 1997; Podcasy and Epperson, 2016), and risk of AD increases post-menopause, when estrogen and progesterone levels are diminished. Could neuroendocrine factors play a role in both ASD and AD incidence? As estrogen is thought to be neuroprotective, what changes occur in Purkinje cells in aging and what is their function in the neuroendocrine system in aging?

## Long-term consequences of atypical cerebellar development

Atypical cerebellar development may result from genetic mutations, many of which have been linked to ASD (Peter et al., 2016; Brady et al., 2020; Van Overwalle et al., 2020). In development, these genetic mutations result in calcium overload and mitochondrial dysfunction, which may result in abnormal brain growth, synaptic plasticity, and connectivity (Zeidán-Chuliá et al., 2014). These genetic mutations can also have long-term consequences on distally connected brain regions, thereby impacting motor, cognitive, and social development as well as the aging brain. Strikingly, 40% of AD-related genes are altered in the cerebellum of autistic patients (Zeidán-Chuliá et al., 2014). Autistic patients develop higher levels of beta-amyloid and are 2.6x more likely to be diagnosed with AD and other dementias (Vivanti et al., 2021). In ASD patients, neural connectivity deficits, excitatory/inhibitory imbalance, and reductions in cerebral gray matter increase risk for development of AD and other dementias (Bodensteiner and Johnsen, 2005; Limperopoulos et al., 2007, 2010; Messerschmidt et al., 2008; Vivanti et al., 2021), while exact mechanisms in aging are less known. There is a need for additional research following ASD patients throughout their lifespan to understand the underlying biological mechanisms that put these patients at higher risk for dementia to determine possible preventative treatments. Future research should also investigate similar cerebellar biological mechanisms between dementias and other neurodegenerative disorders, such as Parkinson's disease (Wu and Hallett, 2013).

## Discussion

In conclusion, the cerebellum has bidirectional connections throughout the brain which may have a large impact on behavior and health across the lifespan. Cerebellar abnormalities can have acute and long-term consequences via modulation of activity in other brain regions, which may be sex dependent. The cerebellar neuroendocrine system is understudied in both development and aging, but there is evidence that Purkinje cells may play a role in sexually differentiating the cerebellar cortex. Understanding these functions could be critical for understanding neurodevelopmental disorders and various aging health challenges, including menopause and dementia. Cerebellar contributions to aging, dementias, and neuropsychiatric disease should receive more attention in clinical settings, for both males and females, and needs to be included in neuroimaging studies, as too often the cerebellum is used only as a reference region (Lacalle-Aurioles et al., 2013; Shigemoto et al., 2018; Young et al., 2021; Leng et al., 2023). There are many unanswered questions. In particular: what biological mechanisms are similar between neural developmental disorders, neurodegeneration, and neuropsychiatric disorders? Do we need novel experimental approaches to answer these questions and are our assays sensitive enough to distinguish sex differences? Lastly, interventions aimed at improving health and disease should include cerebellar function and these cerebellar-cerebral pathways might be a possible treatment avenue with novel targets not yet explored.
